# MicroRNAs Associated With a Good Prognosis of Acute Myeloid Leukemia and Their Effect on Macrophage Polarization

**DOI:** 10.3389/fimmu.2020.582915

**Published:** 2021-01-15

**Authors:** Alexandra Neaga, Cristina Bagacean, Adrian Tempescul, Laura Jimbu, Oana Mesaros, Cristina Blag, Ciprian Tomuleasa, Corina Bocsan, Mihaela Gaman, Mihnea Zdrenghea

**Affiliations:** ^1^Department of Hematology, Iuliu Haţieganu University of Medicine and Pharmacy, Cluj-Napoca, Romania; ^2^Department of Hematology, Brest University Medical School Hospital, Brest, France; ^3^U1227 B Lymphocytes and Autoimmunity, University of Brest, INSERM, IBSAM, Brest, France; ^4^Department of Pediatrics, Iuliu Hatieganu University of Medicine and Pharmacy, Cluj-Napoca, Romania; ^5^Department of Hematology, Ion Chiricuta Oncology Institute, Cluj-Napoca, Romania; ^6^Department of Pharmacology, Toxicology and Clinical Pharmacology, Iuliu Haţieganu University of Medicine and Pharmacy, Cluj-Napoca, Romania; ^7^Department of Hematology, Carol Davila University of Medicine and Pharmacy, Bucharest, Romania

**Keywords:** acute myeloid leukemia, non-coding RNAs, macrophages (M1/M2), macrophage polarization, tumor suppressor microRNA

## Abstract

Acute myeloid leukemia (AML) is an aggressive myeloid malignancy with poor outcomes despite very intensive therapeutic approaches. For the majority of patients which are unfit and treated less intensively, the prognosis is even worse. There has been unspectacular progress in outcome improvement over the last decades and the development of new approaches is of tremendous interest. The tumor microenvironment is credited with an important role in supporting cancer growth, including leukemogenesis. Macrophages are part of the tumor microenvironment and their contribution in this setting is increasingly being deciphered, these cells being credited with a tumor supporting role. Data on macrophage role and polarization in leukemia is scarce. MicroRNAs (miRNAs) have a role in the post-transcriptional regulation of gene expression, by impending translation and promoting degradation of messenger RNAs. They are important modulators of cellular pathways, playing major roles in normal hematopoietic differentiation. miRNA expression is significantly correlated with the prognosis of hematopoietic malignancies, including AML. Oncogenic miRNAs correlate with poor prognosis, while tumor suppressor miRNAs, which inhibit the expression of proto-oncogenes, are correlated with a favorable prognosis. miRNAs are proposed as biomarkers for diagnosis and prognosis and are regarded as therapeutic approaches in many cancers, including AML. miRNAs with epigenetic or modulatory activity, as well as with synergistic activity with chemotherapeutic agents, proved to be promising therapeutic targets in experimental, pre-clinical approaches. The clinical availability of emerging compounds with mimicking or suppressor activity provides the opportunity for future therapeutic targeting of miRNAs. The present paper is focusing on miRNAs which, according to current knowledge, favorably impact on AML outcomes, being regarded as tumor suppressors, and reviews their role in macrophage polarization. We are focusing on miRNA expression in the setting of AML, but data on correlations between miRNA expression and macrophage polarization is mostly coming from studies involving normal tissue.

## Introduction

Acute myeloid leukemia (AML), the most common form of acute leukemia in adults, is a heterogeneous myeloid malignancy. It is characterized by an accumulation of immature blast cells due to deficient maturation, uncontrolled proliferation and prolonged survival of a myeloid progenitor. The backbone of intensive treatment approaches is unchanged for almost half a century, and improvements in long-term survival of patients are unimpressive. There is tremendous interest in the development of new therapeutic approaches, including the identification and subsequent validation of new therapeutic targets (1–5).

MicroRNAs (miRNAs) are part of a recently described mechanism of post transcriptional modulation of gene expression. They act inhibiting translation and speeding up degradation of target messenger RNAs by an imperfect pairing with the 3’-untranslated region (3’-UTR) (6, 7).

miRNAs play an important role in regulating normal haematopoiesis: myeloid differentiation, cell cycle, proliferation, apoptosis, and gene methylation (2–4). In hematological malignancies starting from hematopoietic stem and progenitor cells, miRNA could function either as tumor-suppressors or as tumor-promoters/oncomiRs (3–5, 8).

The tumor microenvironment is credited with a critical role in cancer pathogenesis. The involvement of stromal macrophages in solid cancer pathogenesis is currently being deciphered, but data on their role in leukemia is scarce (9).

## Tumor Associated Macrophages, Phenotypes, and Regulation Factors

Macrophages are the sentinels of the innate immunity. They were first described in the 1880s by Metchnikoff, who also observed that these peculiar cells recognize, engulf and destroy the pathogens, the senescent cells or other debris, thus describing the phagocytosis process (10). Later, it was discovered that apart from their role in the initiation of inflammatory responses, macrophages are involved in tissue repair and remodeling too (11). There are two types of macrophages: monocyte derived macrophages, that evolve from the blood monocytes, by migrating to tissues and becoming macrophages in inflammatory conditions, and tissue-derived macrophages, that emerge during the gestation period from the yolk sack (12). Both are terminally differentiated, effector cells.

In response to epigenetic, microenvironmental and extrinsic factors, macrophages become activated, acquiring new phenotypes, such as the “classical” pro-inflammatory one and the “alternative” anti-inflammatory one (13, 14). Macrophage polarized activation has profound effects on immune and inflammatory responses, but the understanding of mechanisms involved is still incipient.

There is malleability in phenotype switching by elements of the environment and feedback mechanisms. Excessive inflammation causes cell and tissue destruction, an underlying cause of many diseases, including cancer. Coincident with the activation of Toll-like receptor (TLR) signaling, a feedback of anti-inflammatory pathways and mechanisms start modulating inflammation until tissue repair is complete (15).

As a parallel to Th1 and Th2 cytokines paradigm, the pro-inflammatory, anti-tumoral macrophages are categorized as M1, whilst those with anti-inflammatory, wound healing, and pro-tumorigenic activity, are referred to as M2 macrophages. An imbalance in M1/M2 activation was described in appearance of various diseases, including cancer. Macrophages get polarized towards the M1 phenotype under the effect of lipopolysaccharide (LPS) and Th1 type cytokines, such as INF-γ or TNF-α and finally express on their surface, among other markers, toll like receptor 2 (TLR-2) and TLR-4. The expression of M1 subtype is regulated by transcription factors, such as the signal transducer and activator of transcription 1 and 5 (STAT-1, STAT-5), nuclear factor kB (NF-kB), interferon regulatory factors 3 and 5 (IRF-3, IRF-5), and hypoxia inducible factor-1 alpha (HIF-1α) (15–17). In response to Th2 type cytokines, like IL-4, IL-13, or IL-10, macrophages are balanced towards an M2 phenotype. Expression of the M2 phenotype is regulated by STAT-6, peroxisome proliferator-activated receptors gamma and delta (PPARγ, PPARδ), IRF4, and HIF-2α. Several variants of M2 phenotypes were described, responding to and releasing different types of cytokines: M2a, M2b, M2c, M2d (18, 19).

Despite the fact that M1 and M2 phenotypes are antagonistic, they are also intimately intertwined. The STAT family, the nuclear receptor PPAR-γ, the cAMP responsive element-binding protein (CREB) - CCAAT/enhancer-binding protein (C/EBP) axis, the interferon regulatory factors (IRF), and the NF-*κ*B family all participate in the regulation of macrophage polarization, involving many signaling pathways, including JAK/STAT, JNK, PI3K/AKT, and Notch. The balance between the activation of STAT1 and STAT3/STAT6, tightly regulates M1/M2 polarization and activity. NF-κB p65/p50 and STAT1 activation promotes M1 polarization, while STAT3 and STAT6 activation by IL-4/13 and IL-10 increases M2 polarization. PPARδ and PPARγ control distinct aspects of M2 macrophage activation and oxidative metabolism. KLF4, downstream of STAT6, participates in the promotion of M2 macrophage functions by suppressing the NF-*κ*B/HIF-1α-dependent transcription. IL-4 induces not only the myelocytomatosis viral oncogene homolog (c-Myc), which controls the expression of a subset of M2-associated genes, but also the M2-polarizing IFN regulatory factor (IRF) 4 axis to inhibit IRF5-mediated M1 polarization. IL-10 promotes M2 polarization through the induction of p50 NF-*κ*B homodimer (NF-*k*B p50/p50), c-Maf, and STAT3 activities. CREB induces IL-10 downstream of p38 activation. The transcription factor CREB is an activator of C/EBPβ expression. C/EBPβ is one among several transcription factors in addition to KLF4, which activates M2 polarization. The CREB-C/EBPβ signaling axis also favours M2 polarization (15, 20).

## Tumor Associated Macrophages in AML

When the balance in M1/M2 polarization is broken, the tumor associated macrophages (TAMs) appear, which, as their name suggests, support and stimulate the tumor cells growth. There are substantial differences between the TAMs and LAMs (leukemia associated macrophages) and not all of them are elucidated (21). They share mostly M2 characteristics, as they are able to heal and nurture the tumor. Such macrophages are also present in the tumor microenvironment of AML and there is a paucity of data on their precise phenotype (20). It is also unclear which elements are accountable for changing the status of macrophage polarization, facilitating the growth of malignant cells, instead of restraining it. There is insufficient data about the interaction of stromal cells and leukemic cells, as opposed to the extensive knowledge about the role of macrophages in solid tumors. The bone marrow microenvironment can be altered by the leukemic stem cells (LSCs) in a way that supports the development of LSCs instead of the hematopoietic stem cells (HSCs), ultimately heading to chemoresistance (9). It is important to underline the fact that *in vivo* activation of macrophages phenotype model in AML is scarce due to complexity and insufficient understanding, but M2-like macrophages are associated with a poorer survival in AML, and the induction of M1 features contributes to extended survival and appears to inhibit the pro-leukemic effect (21).

## miRNA Correlation With Macrophage Phenotypes Under Physiologic Conditions

Despite miRNAs being recognized as highly conserved across species, there are a lot of divergent results between human and mouse studies investigating the association of miRNA expression to macrophage phenotypes (22). In the following sections, we chose to refer mostly to human studies.

*In vitro* studies of polarized macrophages were performed to establish the miRNA profile of M1 versus M2 macrophages. Recent studies suggest that miRNA activity could control macrophage reaction to environmental signals. Macrophages and their precursors can be recruited by tumors to create a nourishing stroma. miRNAs appear to be involved in the regulation of all phases of macrophage development and proliferation, both under normal conditions or in cancer (23). Below we discuss the reported roles of miRNAs in the setting of macrophage polarization towards M1 or M2 phenotype and key regulators known to control macrophage development, recruitment, differentiation, and M1 or M2 polarization.

**miR-22-3p** was found to be increased in human M2c macrophages (24, 25).

In human MDMs, miR-26a-2* was decreased in both M1 and M2b, and slightly increased in M2c and miR-26a-2 was found in M2c macrophages (20). miR-26a/miR-26b induced a pro-inflammatory M1 polarization by downregulating macrophage colony-stimulating factor (M-CSF) through the PI3K/Akt pathway and IL-10 expression and increasing innate interferons (26).

In human MDMs, miR-29b-1* and miR-29b were found in M1 (20, 24, 25) and in M2b macrophages (20).

**MiR-34a** promotes M2 polarization (27).

**miR-124 (124-1, 124-2, 124-3)** was increased in M2b-polarised cells and seems to be a suitable marker for the M2-like macrophages and a therapeutic target to alter M1/M2 equilibrium (28). miR-124 decreases STAT3 and proinflammatory cytokines, promoting M2 polarization (27, 29).

**miR-125a-5p** expression was amplified after monocyte polarization under M2b (30) or M1, M2c conditions (24, 25). It has been recently shown that monocyte miR-125a-5p expression is enhanced when cells are polarized toward M2-alternatively activated macrophages with immunoregulatory phenotypes, particularly IL-10-producing M2b (30).

**miR-146a** was reported to be increased in human M1 polarised macrophages (24, 25). Also, miR-146a suppressed M1 and promoted M2 polarization, enhanced the activation of M2 macrophage and increased the expression of IL-10 through Notch1-dependent mechanism and by increasing PPARγ in murine macrophage RAW264.7 (31).

**miR-193b** was found increased in M1and in M2a (20, 24, 25). IL-4 treatment increased the accumulation of miR-193b during human monocyte-to-macrophage differentiation (20, 32, 33).

**Figure 1:**
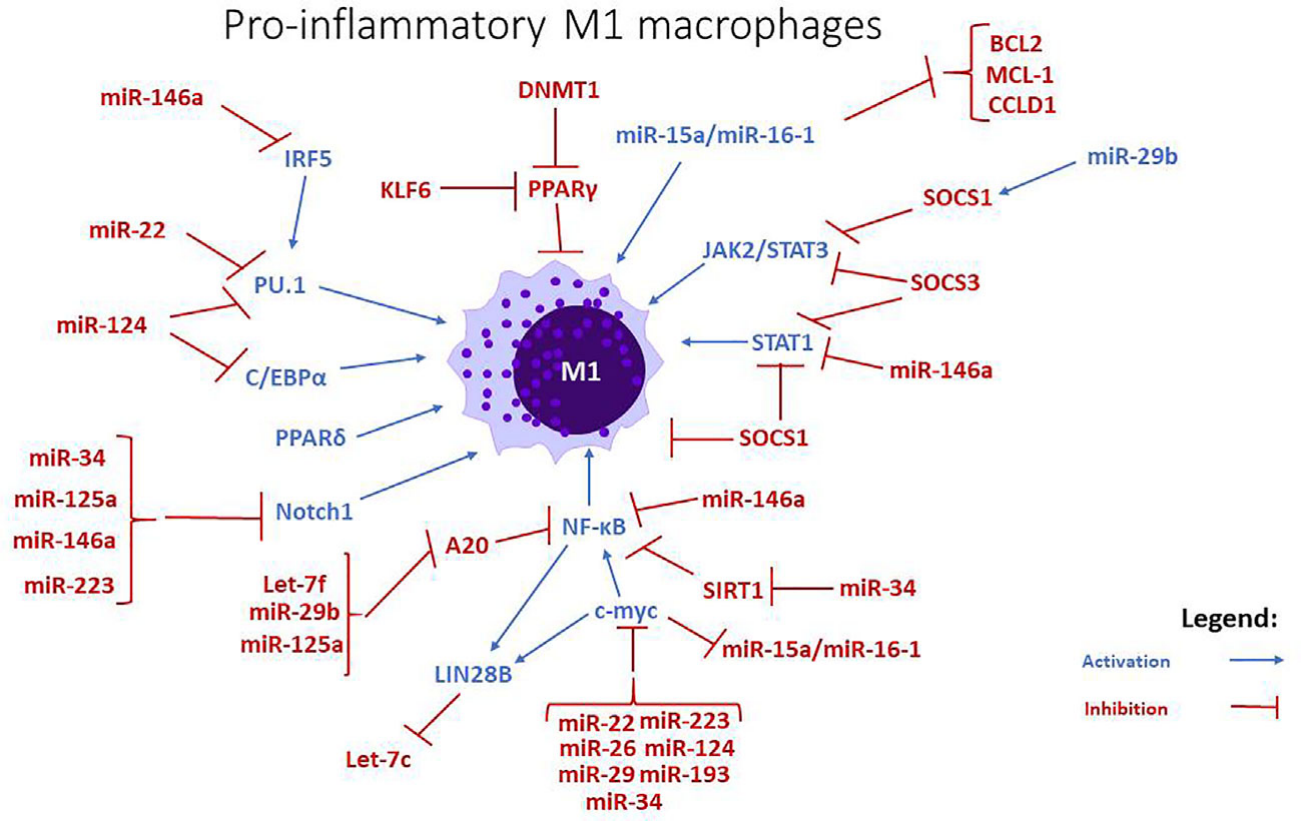
miRNAs and signaling pathways involved in the regulation of pro-inflammatory M1 macrophages. miRNAs like let7-f, miR-15a/-16, miR-22, miR26, miR-29b, stimulate the differentiation towards an M1 phenotype, while miR-34, miR-125a, miR-146a, and miR-223 are negative regulators. The action of miR-193 is uncertain, but some studies imply that it mostly stimulates the polarization towards M2 phenotype.

**Figure 2:**
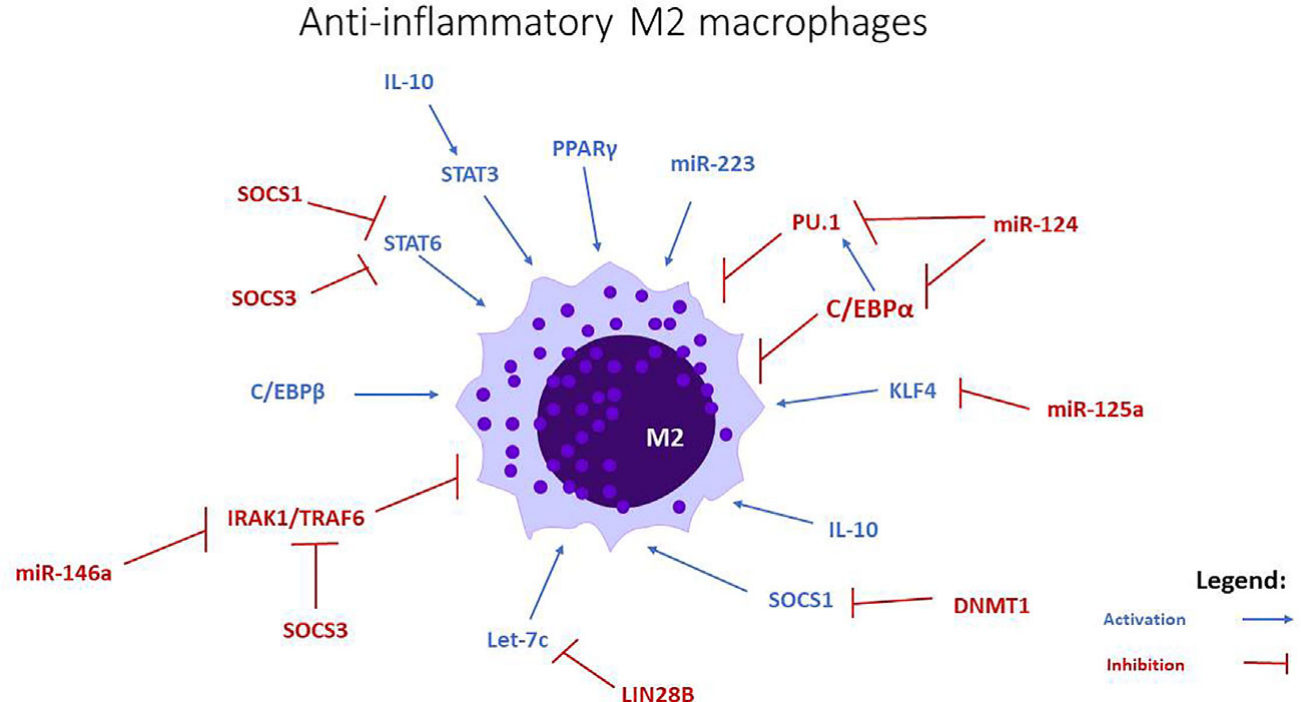
miRNAs involved in M2 anti-inflammatory macrophage polarization. Let-c, miR-34a, miR-124, miR-125a, miR-146a, and miR-223 stimulate the polarization towards this phenotype, through the modulation of transcription factors and intracellular signaling pathways.

**miR-223** promotes M2 polarization (27) *via* targeting anti-inflammatory SOCS1, CEBP-β, and PPARγ. Rasa1 and Nfat5 are miR-223 targets that are important for PPARγ-dependent macrophage alternative activation. miR-223 also inhibited proinflammatory regulator Pknox1 which regulates macrophage classical activation (34) (**Figures 1** and **2**).

The potent anti-inflammatory cytokine IL-10 is important for attenuating the inflammatory response (13, 35). IL-10, *via* STAT3, suppresses mTOR activity through the induction of an mTOR inhibitor, DDIT4 (35). IL-10-STAT3-DDIT4 is important for the inhibition of mTORC1, and the activation of adenosine monophosphate-activated protein kinase (AMPK) by IL-10 also contributes to mTOR inhibition (35). IL-10 mRNA and protein levels in bone marrow-mononuclear cells (BM‐MNCs) were higher in AML patients as related to healthy volunteers, along with IL-10 receptors and the adhesion molecule E-cadherin (36). IL-10 is capable of up-regulating miRNAs, which contributes to the anti-inflammatory response, like oncomiR miR-146b, and down-regulating those that are highly pro-inflammatory, such as miR-155 or miR-9 (37).

## miRNAs Associated With Good Prognosis of AML and Their Potential Therapeutic Targets

Tumor suppressor miRNAs are repressing the expression of genes involved in various oncogenic signaling pathways that are necessary for basic cancer cell survival, as well as cancer stemness, metastasis, and chemoresistance. Epigenetic silencing of tumor suppressor genes frequently occurs due to aberrant DNA hypermethylation in cancer cells. Numerous tumor suppressor miRNAs such as miR-22, miR-29, miR-34a associated with a good prognosis, and long-term survival for AML patients have been found to be decreased in AML (**Table 1**). In the following section we will focus on the most important tumor suppressor miR, their role in AML and their potential as therapeutic targets.

**Table 1:** Tumor suppressor miRNA targets in AML miR.

	Macrophage phenotype expressing the miR	Promoted macrophage phenotype	Downregulated targets
let-7f	M1, M2	M1	A20/TNFAIP3, IL-10 (38)
miR-15a/-16		M1	IKKα, CCND1, BCL-2, MCL- 1, WT1, PD-L1 (39)
miR-22	M2c	M1	c-myc, HOXA7, FL3, CRTC1, PU.1 (40)
miR-26a/-26b	M2c	M1	c-myc, E2F1, E2F7, CCND2, TET1 (41)
miR-29b	M1, M2b	M1	c-myc, MCL-1, KIT, FL3, DNMT, AKT2, CCND2, SP1, CDK6 (42)
miR-34a	M2c	M2	c-myc, PD-L1, HMGB1, CREB, BCL2, SIRT (43)
let-7c	M2a	M2	C/EBP-δ, PAK1, PBX2 (44)
miR-124	M2b	M2	c-myc, CDK4/CDK6, EZH2, C/EBP-α, PU.1, STAT3 (45)
miR-125a	M1/M2b/M2c	M2	C/EBP-δ, KLF4, SIRT7 (39)
miR-146a	M1	M2	IRAK1/2, TRAF6, CXCR4, MyD88, Notch (46)
miR-193	M1, M2a	M2?	c-myc, c-kit, CCND1, AML1/ETO, DNMT3, KRAS, SOS2, HDAC3 (47)
miR-223		M2	c-myc, E2F1, FBXW7, IKKα, STAT3, Pknox1, PU.1, Notch (48)

### Let-7

Let-7 *(*lethal-7), one of the initial miRNA families to be discovered, is composed of let-7a-1, let-7a-2, let-7a-3, let-7b, let-7c, let-7d, let-7e, let-7f-1, let-7f-2, let-7g, let-7i, and miR-98. It was recently shown that the *let-7* family members tend to act as tumor suppressors by supressing oncogenes such as RAS, c-MYC, BCL-X_L_, NF-*k*B, and High Mobility Group A2 (HMGA2) in various cancers (49). They also promote differentiation during development. Expression of tumor suppressor let-7 is decreased in a variety of tumors and one of the cause of the global post-transcriptional downregulation of the let-7 miRNA family is the activation of either LIN28A or LIN28B, two highly related RNA binding proteins and proto-oncogenes (49). Elevated levels of LIN28B protein in bone marrow samples independently associate with worse survival in AML patients (50).

let-7c targets C/EBP-δ, a transcriptional factor that plays a significant role in inflammatory reactions (51, 52). Let-7c also targets serine/threonine kinase family member p21-activated kinase (PAK)1 and pre-B cell leukemia transcription factor 2 (PBX2). The upregulation of enhancer of zeste homolog 2 (EZH2) determines the loss of let-7c, PAK1 elevation and human M1 polarization *via* NIK-IKK-NF-*k*B signaling (53). PML/RARα-positive blasts from acute promyelocytic leukemia (APL) patients display lower levels of miRNA let-7c than in regular promyelocytes. This expression increases after all-trans-retinoic acid (ATRA) treatment (44). The ectopic expression of let-7c promotes granulocytic differentiation of AML cell lines and blasts. PBX2, a well-known homeodomain protein whose abnormal expression enhances homeobox A9 (HOXA9)-dependent leukemogenesis, is a new let-7c target that may impact the AML phenotype, suggesting that that perturbation of the let-7c-PBX2 pathway may have a beneficial role in AML (44).

let-7f targets A20/TNFAIP3, a feedback inhibitor of the NF-*κ*B pathway that was found in mouse Mycobacterium tuberculosis-infected macrophages (54). let-7f may also target some genes, such as PRKAA2 (AMPK, AMP-activated protein kinase), CCNJL (cyclin J-like), MBD2 (methyl-CpG binding domain protein 2), CYP19A1 (38). However, it was discovered that Let-7f is downregulated in t(11q23) AML versus all other AML patients (39). Dai et al. study reported a reduced expression of let-7f in primary leukemia cells from patients with refractory AML and in adriamycin (ADM)-resistant leukemic cell line, K562/A02 (38). Interestingly, this suggests that let-7f may serve as a tumor suppressor. The enhanced let-7f expression *in vitro* could make sensitive K562/A02 cells to ADM, showing that down-regulation of let-7f is implicated in the acquisition of drug resistance in AML (38, 39).

### miR-15a/miR-16-1

The miR-15a/16-1 cluster is believed to function as a tumor suppressor, because its validated gene targets are important oncogenes. Genes which regulate the cell cycle control cell outcome and induce apoptosis by targeting multiple oncogenes, including *BCL2*, *Cyclin-D1 (CCLD1)*, Myeloid Cell Leukemia-1 (*MCL-1)*, and others (55, 56). In one study, an important inverse correlation between miR-15a or miR-16-1 expression and Wilms’ tumor 1 (WT1) oncogene protein levels in primary AML blasts was found (55). It was also established that miR-15a was downregulated in patients with t(11q23) versus all other AML patients (39).

Additionally, miR-16 was found to be down-regulated in FLT3-ITD positive murine myeloid FDC-P1 cells and FLT3 inhibition tends to correct this down-regulation, therefore suggesting a tumor suppressor activity in FLT3/ITD-mediated leukemic transformation (57, 58).

Overexpression of the chemokine stromal-derived factor 1 α (SDF-1/CXL12) and its receptor CXCR4 is a hallmark of many hematological malignancies, including AML, and generally correlates with a poor prognosis. CXCR4 is important in the preservation and survival of acute AML blasts in the bone marrow microenvironment. In AML patients, hypoxia induced CXCR4 expression is associated with poor prognosis, not depending on the presence of the mutated *FLT3* gene. FLT3-ITD mutations trigger CXCR4 signaling and are associated with increased CXCR4 expression in primary AML cells. However, it was demonstrated that an antagonist of CXCR4, BL-8040, determined the apoptosis of AML cells *in vitro* and *in vivo* and induced the robust mobilization of AML blasts from the bone marrow (BM). Apoptosis was mediated by the up-regulation of miR-15a/miR-16-1, resulting in down-regulation of the target antiapoptotic genes BCL2, MCL-1, and cyclin-D1 in MV4-11 and U-937 leukemic cell lines. Interestingly, leukemic cell death was produced directly by the overexpression of miR-15a/miR-16-1. BL-8040-induced apoptosis was also facilitated by the inhibition of survival signals through inhibition of AKT/ERK pathways. Notably, treatment with a BCL2-inhibitor induced apoptosis and combined with the CXCR4 antagonist BL-8040 enhances cell death, making this mechanism a very attractive target (56, 58, 59).

In a recent phase 1b clinical trial, the combination of a second generation CXCR4 antagonist, LY2510924, with idarubicin and cytarabine (IA) was reported to be safe in the relapsed/refractory (R/R) setting. However, LY2510924 at dose of 10 and 20 mg/day suppresses CXCR4 receptor blockade in some, but not all patients. The FDA has allowed a higher dose of 30 mg of LY2510924 in a new clinical trial (60).

Beside the developments mentioned above, there are ongoing clinical trials of miR-16 mimics -TargomiRs, which are minicells (EnGeneIC Dream Vectors) loaded with miR-16-based mimic miR- which successfully completed phase 1 trials and will soon start phase 2 clinical investigation in malignant pleural mesothelioma. Due to these advancements, miR-16 mimics could be evaluated in treating hematological malignancies, including AML (56, 61).

### miR-22

miR-22 has an important role in monocytic differentiation in healthy and leukemic cells. miR-22 expression is increased during monocyte/macrophage differentiation of HL-60, THP1 leukemia cells lines, and CD34^+^ hematopoietic stem/progenitor cells (62).

Studies showed that miR-22 was down-regulated in low miR-188–5p expressers cytogenetically normal acute myeloid leukemia (CN-AML). This fact was associated with longer overall survival (OS) and event free survival (EFS) (63) in *de novo* AML (40). One of the causes of miR-22-downregulation in AML is TET1/growth factor independent 1 (GFI1)/EZH2/SIN3A-mediated epigenetic repression and/or DNA copy-number loss (40). miR-22 inhibited multiple oncogenes, including CREB-regulated transcription coactivator 1 (CRTC1), homeobox (HOXA)7, FLT3 and MYCBP (a MYC-binding protein), and consequently the CREB and MYC pathways, leading to the inhibition of leukemia progression *in vivo* (40).

Down-regulation of miR-22 levels and E26 transformation-specific family transcription factor (PU.1) were reported in AML patients (62). miR-22-mediated MECOM (MDS1 and EVI1 complex locus protein EVI1) degradation increased c-Jun levels, which interacts with PU.1 transcription factor to promote monocyte/macrophage differentiation (62). Consequently, the reintroduction of miR-22 inhibited the growth of AML bone marrow blasts, suggesting that the restoration of miR-22 expression or function could have a therapeutic potential to treat AML (62). These studies suggest that miR-22 could be a promising potential marker in diagnosis and treatment monitoring, and a prospective therapeutic target in AML (40, 62).

### miR-26a/miR-26b

The initial *in vitro* and *in vivo* studies revealed that miR-26a/b can function as an oncogenic miRNA, being associated with unfavourable OS in 53 patients with AML (64). The tumor suppressor ten-eleven translocation (TET) 2, a member of the TET methylcytosine dioxygenase family, is frequently mutated and under extensive miR regulation in hematopoietic malignancies. It was suggested that *TET2 gene*-targeting miRs, such as miR-26a/b, may play oncogenic roles in hematopoietic cancers (65). However, miR-26 has been incriminated to target cyclin-D2 (CCND2) and cyclin-E2 (CCNE2), consequently inhibiting the cell cycle in malignant cells (66).

The expression of miR-26a was down-regulated in primary blasts from AML patients (41, 67). In myeloid leukemia cells, c-Myc enforces transcriptional repressor E2F1 activity and contributes to increased EZH2 levels (41). During myeloid differentiation of AML cells, miR-26a induction was associated with a decrease in c-Myc and EZH2 levels (41, 67). miR-26a interfering with E2F7 (which promotes cell cycle progression and inhibits monocytic differentiation of AML cells), leads to the inhibition of c-Myc activity and downregulation of the oncogenic miR-17-92 cluster (67).

In another study, miR-26a-5p was also reported to be decreased in AML patients and subsequently the peroxiredoxin III (PrxIII), a reactive oxygen species (ROS) scavenger, was increased (68). Increased levels of PrxIII caused decreases in ROS levels in AML samples, which are required for hematopoietic stem cell differentiation (68).

### miR-29b

miR-29b family consists of three different isoforms (miR-29a, miR-29b, miR-29c) and it’s downregulated in different myeloid and lymphoid neoplasms and solid cancers (69). Compared with control groups miR-29 family had lower expression in different subtypes of AML (39, 70–73). miR-29a, miR-29b, and miR-29c were downregulated in PBMCs and bone marrow CD34^+^ cells in AML patients as opposed to healthy donors (33, 42, 74, 75) miR-29b targeted DNA methyltransferases (DNMTs) DNMT3A and DNMT3B, by reducing the global DNA methylation and specificity protein (SP)1) and DNMT1, by targeting SP1 and enhancing tumor cell chemosensitivity (76, 77). The ectopic transfection of synthetic miR-29b into leukemia cells revealed that miR-29b targets genes connected to apoptosis (MCL-1, TRAF4, and MYBl2), cell cycle (CDK4, CDK6, and CCND2), and cell proliferation (JAK2 and IGF1) (70). The overexpression of miR-29b was inversely correlated to MCL-1 in 45 primary AML samples (39, 70–73).

However, in some studies, miR-29b has been linked to unfavourable survival rates of AML patients (64, 78). In a cohort of CN-AML, multiple tumor suppressor *TET2*-targeting miRs, such as miR-29b/-29c and miR-125b, were preferentially overexpressed in *TET2*-wildtype samples than those with *TET2*-mutations (65). *In vivo* expression of TET2-targeting miRNAs generated hematopoietic cell expansion and/or myeloid differentiation bias, and co-expression of TET2 fixed these phenotypes. Cheng et al. suggested that increasing *TET2* expression could be a likely approach to combat certain groups of hematopoietic cancers (65).

In another study, miR-29b expression level was inversely related to MLLT11 expression in AML patients and AML patients with low miR-29b and elevated MLLT11 expression had poor OS (71). The decreased miR-29 expression in AML blasts was correlated with increased anti-apoptotic molecule AKT2, cell cycle regulation cyclin-D2, and Myc levels (42). PI3K/AKT pathway is a negative regulator of miR-29b and inversely, miR-29b reduces AKT phosphorylation (42, 79).

The nuclear factor erythroid 2-related factor 2 (NRF2), a transcription factor which protects cells from the oxidative damage, was reported to be constitutively expressed in AML and promoted leukemic cell survival (80). The activation of Nrf2 is correlated with decreased levels of miR-29b and associated with treatment resistance to regular chemotherapy, as well as with reduced survival of cancer patients. The transfection with miR-29b mimic of primary AML samples (either alone or co-treated with standard AML chemotherapy) resulted in increased cell death responsiveness (80).

Another transcription factor, MYC, activated by c-kit, binds and inhibits the miR-29b promoter (81). The decrease in miR-29b expression was correlated with upregulation of SP1 levels, subsequently allowing the formation of the SP1/NF-*k*B complex. This complex binds regulatory structures to increase c-kit expression and the formation of a complex with histone deacetylase 1 (HDAC1), which promotes the down-regulation of miR-29b expression (81, 82). Remarkably, the proteasome inhibitor bortezomib, acting through a miR-29b-dependent mechanism, downregulated SP1 and decreased global DNA methylation. Consequently, it induced M2 macrophage marker suppressor of cytokine signaling-1 (SOCS-1) promoter demethylation and protein up-regulation, reduced proliferation, and increased apoptosis (83). These promising data suggest that AML patients may benefit from histone-deacetylase inhibitors (HDACI) therapy. Studies of cytogenetic, molecular and *in vitro* growth features of primary blasts related to HDACI response will deliver understanding into how to select AML patients that are prone to respond to HDACIs (82, 84).

On the other hand, treatment with type I IFN-β supressed miR-29b during human monocyte-to-macrophage differentiation *in vitro* (33). mir-29 expression can be inhibited by c-Myc, hedgehog signaling, and inflammatory pathways (TLR ligation, activation of NF-*k*B), commonly activated in cancer (74). C/EBPα is an activator of miR-29a/b (75).

In a study of 53 older AML patients with previously untreated disease, who were not suitable candidates for chemotherapy, or who refused it, higher miR-29b pre-treatment expression was correlated with improved response to decitabine (a DNA methyltransferase inhibitor) and better outcome in AML (85). The HDACI AR-42 increased miR-29b levels and led to downregulation of known miR-29b targets (SP1, DNMT1, DNMT3A, and DNMT3B). Importantly, the anti-leukemic activity *in vitro* and *in vivo* of decitabine was increased, when HDACIs were given before decitabine, as decitabine traditionally is given before HDACIs (82). This could be explained by the increased miR-29b levels by HDACI AR-42, which will target DNMT expression, consequently decitabine better inhibiting the activity of remaining DNMT. Alternatively, lower levels of SP1 induced by increased *miR-29b* expression leads to a reduced transcription of genes, known to promote AML leukemogenesis, such as modified and/or upregulated receptor tyrosine kinases (i.e., FLT3 and KIT) (81, 86).

Conversely, the transferring-conjugated anionic lipopolyplex nanoparticle synthetic *miR-29b* mimics Tf-NP-*miR-29b* was shown to downregulate DNMTs, cyclin-dependent kinase (CDK) 6, SP1, KIT, and FLT3, to suppress AML growth, to impair colony formation, and to reduce cell viability *in vitro* in AML patient samples (87). In addition, priming AML cells with Tf-NP-miR-29b before therapy with decitabine resulted in striking decrease in cell viability and growth, showing improved antileukemic action, compared to *in vivo* single-agent decitabine treatment (87).

### miR-34

miR-34 family, consisting of miR-34a, -34b, and -34c plays a key role as tumor suppressor miR. MiR-34a was the primary recognized tumor suppressor gene which is downregulated in AML (88).

mir-34 exerts its growth inhibition on cancer cells by repressing HDAC1 (89), targeting factors required for G1/S transition (c-MYC, CDK6), anti-apoptotic proteins (Bcl2, sirtuin protein-SIRT1), proteins involved in invasion (c-MET) (90), and induction of sequential down modulating of Erk/Akt activity (91).

Wang et al. reported that PD-L1 was overexpressed in AML samples and that there was an inverse correlation between PD-L1 and miR-34a gene expression (92). PD-L1 is regulated by p53 *via* miR-34 (93). Overexpression of miR-34a in HL-60 and Kasumi-1 cells blocked PD-L1 expression and reduced PD-L1 surface expression (92). Surface expression of PD-L1 produced by IFN-γ was also reversed by miR-34a. miR-34a transfection diminished the production of PD-L1 downstream of IL-10 and decreased PD-L1-specific T cell apoptosis (92).

The transcription factor, CCAAT enhancer binding protein alpha (C/EBPα) is an activator of miR-34a (3). The CEBPA (which is crucial for granulopoiesis) gene mutations are reported in 8-10% of patients with AML (3). Study of AML samples with CEBPA mutations described a lower expression of miR-34a and increased levels of E2F3, as well as E2F1, a transcriptional target of E2F3. miR-34a influences granulocytic differentiation of AML blast cells with CEBPA mutations. miR-34b was downregulated in patients with t(11q23) AML (39, 88).The high-mobility group box-1 (HMGB1) functions as an anti-apoptotic protein in leukemia, inhibiting cell apoptosis and inducing cell growth. Lower expression of miR-34a correlated with greater expression of HMGB1 in HL-60 and THP-1 cells compared to that in HS-5 cells (43). Higher expression levels of miR-34 and lower expression levels of HMGB1 both considerably promoted apoptosis, inhibited autophagy in HL-60 and THP-1 cells and repressed chemotherapy-induced autophagy by stimulating the autophagy marker LC3 conversion (43). By promoting cell apoptosis and inhibiting autophagy *via* targeting HMGB1, miR-34a may be a potential promising molecular target for AML therapy. Enhanced expression of miR-34a in TIM3 positive leukemia stem cells (LSC) from AML children inhibits the clone expansion, progression and metastasis of leukemia, suggesting that miR-34a is a key regulator which could be taken into consideration as a novel therapeutic agent against LSC (94).

In addition, mir-34b is a negative regulator of CREB expression in myeloid cell lines by binding directly to the 3′UTR of CREB mRNA. In AML cells, the promoter of miR-34b is highly methylated, leading to miR-34b down-regulation in the majority of patients and, consecutive overexpression of the proto-oncogene CREB (88). Additionally, low miR-34b and high CREB expression levels stimulated abnormal myelopoiesis through CREB-dependent pathways *in vitro* and *in vivo* in animal studies, consequently leading to myeloid cell proliferation (88).

miR-34c plays an important role in tumorigenesis, but its role in AML is not well known. A study comparing 122 patients with *de novo* AML with healthy patients showed downregulated miR-34c in AML in the first group (95). However, in another study low expression of miR-34c is associated with dismal prognosis and poor response to treatment (96).

It is important to point out that miR-34 mimic did not induce side effects in mice (97) and was considered to be the first miR mimics to reach the clinic. Therapeutic delivery of a mimic of naturally occurring miR-34, MRX34, promoted tumor-infiltrating lymphocytes and reduced CD8^+^PD1^+^ cells *in vivo* (93). Furthermore, MRX34 plus radiotherapy increased CD8^+^ cell numbers more than therapy alone, and miR-34a delivery reduced the numbers of radiation-induced macrophages and T-regulatory cells (93). Still, a clinical trial of MRX34 (NCT01829971) in refractory advanced solid tumors (such as histologically confirmed viral related hepatocellular, lung cancer, melanoma, ovarian, bladder cancer, sarcoma) was stopped by Mirna Therapeutics, following multiple immune-related severe adverse events (5 cases). It remains to see if the miR-34a replacement combined with other anticancer agents could be a valuable therapeutic option (51, 95, 96).

### miR-124

*In vitro* and *in vivo* studies showed that miR-124 (124-1, 124-2, 124-3) has several molecular targets, including STAT3, proinflammatory cytokines, cyclin-dependent kinase CDK4, CDK6, EZH2, C/EBP-α, and PU.1 (27, 29, 98). miR-124 is a potent tumor suppressor pro-apoptotic miR in several cancers. Downregulation of miR-124 by promoter hypermethylation has been observed in several types of malignancies (99). The promoter of miR-124-1 was homozygously methylated in 15% of AML samples and unmethylated in normal controls (100).

Chen et al. reported that in AML miR-124-1 is underexpressed in 36% of AML patients and is better expressed in the patients with t(15;17) than in others (62% versus 30%) (101). However, there was a trend in AML patients with reduced miR-124-1 to have longer OS and RFS than those without miR-124-1 underexpression (101).

miR-124 expression is inhibited in myelodysplastic syndrome (MDS) and is upregulated in response to epigenetic treatment (EGT) (45, 102). The upregulation of miR-124 expression in response to single-agent EGT with either azacytidine (AZA) or the histone deacetylase inhibitor panobinostat (LBH589) reported *in vitro* in the HL60 leukemic cell line (45) resulted in inhibition of CDK4, CDK6 and EZH2 expression. Accordingly, combination with EGT led to a significant and additive inhibitory effect. PBMCs from patients which responded to the combination EGT for high risk MDS or AML demonstrated *in vitro* significant induction of miR-124 and inhibition of CDK4 and CDK6 (and a trend to inhibit EZH2) mRNA expression. These findings suggest miRNA-124 is a potential biomarker of early response to EGT in myeloid malignancies and a possible therapeutic target (45).

### miR-125a-5p

*miR-125* family consists of miR-125a and miR-125b and regulates hematopoiesis (2). Both miR-125a and miR-125b are overexpressed in different lymphoid and myeloid malignancies (2, 103), but both are also present in solid cancers (104). Different studies suggest that 15%–25% of AML cases overexpress miR-125b. In a knock-in mouse model study, miR-125b was associated with leukemogenesis –via MLL-AF9 – and suppressed apoptosis (2). The highest expression of miR-125a found was in hematopoietic stem cells and expression gradually decreases with differentiation (103). In solid cancers, downregulation of miR-125a is associated with higher invasion, lymph node metastasis and tumor size. In AML low expression of miR-125a is associated with decreased blast count, higher count of leukocytes, platelets, monocytes and promonocytes and with M5 and M3 FAB subtypes (104). The action of both IFN (IFN-β or IFN-γ) and IL-4 consisted of repressing miR-125a-5p accumulation during human monocyte maturation to macrophages (33) and IL-4-dependent repression in alternative activated macrophages occurred *via* the IL-4Rα-STAT6 signaling pathway (105). m*iR-125a-5p* and *miR-125b* have been reported to down-regulate oncogenic SIRT7, a member of the sirtuin family of NAD^+^-dependent histone/protein deacetylase (106).

In a study of 122 newly diagnosed AML samples Garzon et al. reported that miR-125a is downregulated (39) compared to healthy donors. In another AML study (without a healthy control group), miR-125a expression was downregulated in CN-AML. miR-125a expression was most decreased in AML with favourable and intermediate prognostic and associated with decreased survival (104). Ufkin et al. demonstrated in their study on leukemia NB4 cells that miR-125a is transcriptionally suppressed by methylation, and decitabine increased both precursor and mature miR-125a (107).

Mahmud et al. suggested the possible use of the tyrosine kinase inhibitor erlotinib in a subset of adult AML patients that had high epidermal growth factor receptor (EGFR) and EGFR phosphorylation levels relative to normal CD34+ cells measured by RPPA. These studies suggest that ErbB inhibitors are promising therapeutic agents for treating miR-125a-low AML or AML patients that have high levels of epidermal growth factor receptor (EGFR) (107). Treatment with either ectopic expression of miR-125a or inhibition of ErbB *via* Mubritinib, a selective inhibitor of ErbB2 phosphorylation, restored miR-125a expression in human acute promyelocytic leukemia NB4 cells with low miR-125a levels. As a result, it causes decreased cell proliferation, cell cycle progression and enhanced apoptosis (33, 39, 87, 105, 107).

### miR-146a

The family consists of miR-146a and miR-146, which negatively mediates inflammation. Its expression, especially the one of miR-146a, is NF-*k*B-dependent and it is induced by TLR-ligands and pro-inflammatory cytokines, like TNFα and IL-1β (108–110). As mentioned above, through negative feedback, miR-146a and miR-146b possess anti-inflammatory effect, preventing immune overreaction and malignant transformation, miR-146a being negatively correlated with genes involved in the inflammatory response, such as CXCR4, TLR4, CCl23, Ltb4r, LYZ, IL8RB, MyD88 (111, 112). First of all, the CXCR4 expression in normal and leukemic monocytic cells is under the post-transcriptional control of miR-146a. HIF-1α stimulates the miR-146a expression, which lowers the level of CXCR4, while low expression of miR-146a in AML was correlated with high expression of CXCR4, which through his ligand CXCL-12, stimulates the cell harboring in the microenvironment of the BM and was found to be overexpressed in 25%–30% of AML cases (46, 113). However, it has been pointed out the fact that the hypoxia-mediated regulation of microRNA-146a is absent in acute monocytic leukemia, thus enhancing the expression of CXCR4 and providing the leukemic cells with chemoresistance (46).

Apart from mediating the CXCR4 expression, miR-146a appears to debilitate the effect of NF-κB, by repressing TNF receptor-associated factor 6 (TRAF6) and IL-1 receptor-associated kinase 1 (IRAK-1), key adaptor proteins of the NF-*k*B (108, 114). It was suggested that down-regulation of miR-146a could promote the progression of AML through TRAF6-mediated induction of NF-*κ*B (114).

In one study of 53 patients with AML, miR-146a expression in bone marrow specimens was inversely correlated with OS (64). However, when compared to the control lot, miR-146a was downregulated in CD34 bone marrow cells isolated from patients with AML with a normal karyotype and even more decreased in patients who presented chromosome 5q deletion (del[5q]) as compared to controls. The 5q deletion is one of the most frequent cytogenetic anomaly found in AML and myelodysplastic syndrome. Re-expression of miR-146a inhibits survival and growth of leukemic cells, suggesting a role in the pathogenesis of AML (115, 116).

It was reported that reduced miR-146a expression in chromosome 5q deletion high-risk (HR) MDS (HR MDS)/AML patients and miR-146a^-/-^ hematopoietic stem/progenitor cells (HSPCs) resulted in TRAF6/NF-*k*B activation. The increased survival and proliferation of HSPCs from miR-146a^low^ HR MDS/AML is sustained by a neighboring haploid gene, SQSTM1 (p62), necessary for TRAF6-mediated NF-*k*B signaling. The disintegration of p62-TRAF6 complex resulted in cell-cycle blockade and apoptosis of MDS/AML cells. However, TRAF6 is needed for hematopoietic stem cell homeostasis in order to maintain a minimum level of IKKβ/NF-*κ*B (115).

Human miR-146a and TRAF-interacting protein with forkhead-associated domain B (TIFAB) reside on chromosome 5q and are co-deleted in ∼80% of del(5q) MDS and AML. miR-146a inhibits TRAF6 protein expression and reduces its mRNA levels and TIFAB inhibits TRAF6 by destabilizing its protein (116, 117). The combined deletion of miR-146a and TIFAB, which are genes associated with del(5q) MDS/AML, resulted in the interdependent augmentation of TRAF6 expression and hematopoietic dysfunction (117).

An intimate link between high miR-146a-5p expression and better overall survival in 21 types of solid cancer was suggested by the first meta-analysis of the prognostic value of miR-146a-5p expression in diverse cancers. No correlation was found between miR-146a and AML, but only one study was included in this meta-analysis. More studies are needed to elucidate the role of miR-146a in AML (64).

### miR-193

miR-193 is a suppressor miR important in controlling the self-renewal and the expansion of HSCs and progenitor cells by directly suppressing the expression of target genes, such as oncoprotein AML1/ETO, DNMT3a, HDAC3, c-kit, cyclin-D1. It also increases indirectly the activity of tumor suppressor PTEN, which negatively regulates PI3K activity (32, 55).

miR-193a levels were oppositely coordinated with the ones of c-kit proto-oncogene levels measured in 27 primary AML samples and 9 leukemia cell lines (47). The reconstitution of the miR-193a expression in AML cells containing c-kit mutation and/or overexpression, generated a significant cutback in c-kit expression at the RNA, as well as the protein levels, the inhibition of cell growth, and an augmentation of apoptosis and granulocytic differentiation. The inhibitor of DNA methylation 5-azacytidine-induced c-kit proto-oncogene reduction and it could be blocked to a certain degree by a miR-193a inhibitor, that will result in the cancellation of the antiproliferative and the pro-apoptotic effects of 5-azacytidine. These data suggest that miR-193a holds an important role in the formation of AML cells, bringing into the light the hope of a new therapeutic approach by upregulating miR-193a expression for c-kit-positive AML (55). miR-193a and PTEN were identified as targets for AML1/ETO and supported their role in the differentiation block of myeloid precursors in t(8;21) leukemias (32). High levels of miR-193a levels will induce G1 arrest, apoptosis, and will restore the differentiation of the leukemic cells differentiation (83). Li et al. associated the downregulation of miR-193a-3p with fusion protein AML1/ETO expressed in hematopoietic cells from patients diagnosed with t(8;21) AML (32).

The high c-kit expression was associated with the downregulation of miR-193a, which was found in patients with t(11q23)and in cytogenetically normal AML (CN-AML) (39, 118). *RUNX1* was also up-regulated in CN-AML patients, as well as gene sets that regulate the cellular cycle and proliferation, particularly *c-kit, FLT3*, *MYCN*, *MYB*, *MYC*, and *CDK6*, *RUNX1*^high^ were associated with poorer OS and EFS of CN-AML patients (118).

More recently miR-193b-3p levels in bone marrow cells were reported to be decreased in adult patients with CN-AML, especially in cases with FLT3 mutations, and the values were higher in the t(15;17) good prognosis subgroup compared to all AML patients (there was no healthy control group). Low expression of miR-193b-3p correlated with significantly worse OS and EFS in adult, as well as in paediatric patients (119). *In vitro*, miR-193b interfered with cell-cycle progression, differentiation and viability of leukemia cell lines and primary AML samples, by targeting key regulators of MAPK signaling (KIT, KRAS, SOS2) and CCND1 (119, 120). The authors suggest that due to its blocking effect on the entire MAPK signaling cascade, the miR-193b agonists would be highly efficient in AML (119).

### miR-223

miR-223 has been reported to be both, an oncogenic regulator of the tumor progression and a tumor suppressor gene, but in AML it seems to act as the later and to be downregulated (48, 121).

*miR*-223 acts normally as an inhibitor regulator of granulocytic proliferation, along with C/EBPα, E2F1, and PU.1 (2, 122, 123) transcription factors, taking part in an autoregulatory negative feedback. During human monocyte-macrophage differentiation (under GM-CSF- or M-CSF-treatment), the expression of the miR-223 was found to be considerably decreased, which led to higher expression of the serine-threonine kinase IKKα (but not IKKβ or IKKγ) in macrophages, which further induced higher amounts of p52. In the context of decreased expression of RelB, a transcription factor found in untreated macrophages, high p52 expression blocked the basal transcription of both canonical and noncanonical NF-*κ*B target genes (124).

It seems that miR-223 is a direct transcriptional target of the acute myeloid leukamia-specific fusion oncoprotein RUNX1/RUNX1T1 (AML1/ETO) (125). miR-223 levels are lower in t(8;21) AML patients than other AMLs. AML blast treatment with miR-223 oligonucleotides, *RUNX1*/*RUNX1T1* inhibitors, or even hypomethylating agents, has shown to increase miR-223 levels and restore cell differentiation (125).

In another study, miR-223 expression was measured in blasts from 115 AML patients, which led to the correlation of low miR-223 expression levels with worse outcome, while higher miR-223 levels were associated with favourable prognosis (121). Furthermore, miR-223 was hierarchically expressed in AML subpopulations, with lower expression in leukemic stem cell-containing fractions (121).

The miR-223 expression was downregulated in mononuclear cells from bone marrow aspirate samples from patients with AML as compared to healthy subjects (48). *In vitro* experiments on AML cells pointed out that miR-223 inhibits cell proliferation and stimulates cell apoptosis and also the fact that miR-223 target, F-box and WD repeat domain containing 7 (FBXW7), binds proteins such as c-myc, Jun, cyclin-E, and Notch leading to their proteasomal degradations (48).

## Conclusions

The onset and progression of malignancy in AML is not only dependant on intrinsic factors, but also on the tumor microenvironment, and macrophages thus play a crucial role in this setting. Tumors are invaded with by large amounts of macrophages expressing several active miRs, and their interfering with TAM activity could reprogram their phenotypes. The interest in the role of miRNA in cancer, including acute myeloid leukemia has seen a recent surge. In this review we focused on miRNA which, due to their tumor suppressor activity, could play a favourable role in adult AML. The available preclinical data suggest that these miRNA could be useful in deciphering the pathogenesis of AML and contribute to the diagnosis and prognositic assessment of AML. To validate the beneficial effect of the suppressor miRNA mimics, clinical trials are expected. The succesful future approach could be a combination of miRNA mimics with inhibitors of oncogenic miRNA and chemotherapy.

## Author Contributions

All authors contributed to conception and design of the study. AN drafted the manuscript. AT, CBa, OM, LJ, CBl, CT, CBo and MG researched and wrote sections of the review. MZ co-ordinated the team and reviewed the sections and final article. All authors contributed to the article and approved the submitted version.

## Conflict of Interest

The authors declare that the research was conducted in the absence of any commercial or financial relationships that could be construed as a potential conflict of interest.

The handling editor declared a past co-authorship with one of the authors CT. The handling editor declared a shared affiliation with several of the authors AN, CT, LJ, OM, CLB, CB, MZ at time of review.
